# Enhancing Genomic Prediction Accuracy for Body Conformation Traits in Korean Holstein Cattle

**DOI:** 10.3390/ani14071052

**Published:** 2024-03-29

**Authors:** Jungjae Lee, Hyosik Mun, Yangmo Koo, Sangchul Park, Junsoo Kim, Seongpil Yu, Jiseob Shin, Jaegu Lee, Jihyun Son, Chanhyuk Park, Seokhyun Lee, Hyungjun Song, Sungjin Kim, Changgwon Dang, Jun Park

**Affiliations:** 1 Department of Animal Science and Technology, College of Biotechnology and Natural Resources, Chung-Ang University, Anseong 17546, Republic of Korea; jungjae.ansc@gmail.com; 2 Korea Animal Improvement Association, Seoul 06668, Republic of Korea; hsmun@aiak.or.kr (H.M.); greatman009@hanmail.net (Y.K.); scpark@aiak.or.kr (S.P.); jskim@aiak.or.kr (J.K.); ucine76@naver.com (S.Y.); jhson@aiak.or.kr (J.S.); chpark@aiak.or.kr (C.P.); sj_2003@naver.com (S.K.); 3 Dairy Cattle Improvement Center of NH-Agree Business Group, National Agricultural Cooperative Federation, Goyang 10292, Republic of Korea; nicejiseob@naver.com (J.S.); asdko123@nate.com (S.L.); kiya0321@gmail.com (H.S.); 4 Animal Breeding and Genetics Division, National Institute of Animal Science, Rural Development Administration, Cheonan 31000, Republic of Korea; jindog2929@korea.kr; 5 Department of Animal Biotechnology, Jeonbuk National University, Jeonju 54896, Republic of Korea

**Keywords:** genomic selection accuracy, Korean Holstein, Bayesian analysis, deregressing estimated breeding value, genome-wide association study

## Abstract

**Simple Summary:**

The Holstein breed is crucial in the dairy industry in Korea. In this study, we focused on the accuracy of genomic predictions for 12 of 24 significant body conformation traits examined in Korean Holstein cattle. Employing various statistical methods and levels of genetic data, we assessed how accurately these traits could be predicted. The prediction accuracy increased notably when both offspring and parental genetic information were considered. We identified 18 key genetic regions, offering valuable insights for future research to identify specific genes related to these traits. This study highlights the potential of advanced genetic tools to improve breeding strategies for Korean Holstein cattle, in particular for enhancing traits with significant economic and health impacts.

**Abstract:**

The Holstein breed is the mainstay of dairy production in Korea. In this study, we evaluated the genomic prediction accuracy for body conformation traits in Korean Holstein cattle, using a range of π levels (0.75, 0.90, 0.99, and 0.995) in Bayesian methods (BayesB and BayesC). Focusing on 24 traits, we analyzed the impact of different π levels on prediction accuracy. We observed a general increase in accuracy at higher levels for specific traits, with variations depending on the Bayesian method applied. Notably, the highest accuracy was achieved for rear teat angle when using deregressed estimated breeding values including parent average as a response variable. We further demonstrated that incorporating parent average into deregressed estimated breeding values enhances genomic prediction accuracy, showcasing the effectiveness of the model in integrating both offspring and parental genetic information. Additionally, we identified 18 significant window regions through genome-wide association studies, which are crucial for future fine mapping and discovery of causal mutations. These findings provide valuable insights into the efficiency of genomic selection for body conformation traits in Korean Holstein cattle and highlight the potential for advancements in the prediction accuracy using larger datasets and more sophisticated genomic models.

## 1. Introduction

In Korea, the Holstein breed is the mainstay of milk and dairy production. The Korean Animal Improvement Association (KAIA) has developed advanced herd management strategies to maximize production efficiency and profitability, with a parallel focus on reducing methane emissions. This includes standardizing appearance and linear assessment to identify superior dairy cattle. Since the 1990s, assessing conformation traits, which are linked to body stature, longevity, and productivity, has been crucial in the global dairy cattle industry [1]. These traits not only influence cow productivity but also its value as a show animal, underscoring a comprehensive breeding strategy that emphasizes health, fertility, and performance within high-yield dairy systems.

Since its introduction by Meuwissen [2], genomic selection (GS), using thousands of single-nucleotide polymorphisms (SNPs), has revolutionized cattle breeding, enhancing the accuracy of estimated breeding value (EBV) in the dairy and beef cattle industries. This improvement has been attributed to the development of various GS models, validation methods, and SNP panels, all of which are aimed at refining genomic prediction accuracy. The effectiveness of GS is predominantly determined by the accuracy of the molecular breeding value (MBV), which is influenced by factors such as the size of the training population, marker density [3,4,5,6], clustering methods for cross-validation, and the relationship between reference and validation populations [7,8,9]. The selection of response variables, like EBV (estimated breeding value) or DEBV (deregressed-EBV), and the incorporation of a weighting factor or a residual polygenic component into the model [10,11] also play pivotal roles. Furthermore, the success of GS is contingent on the genetic architecture [12,13,14] and population structure [4,15].

To enhance the profitability of dairy cows, maximization of milk production, maintenance of good health status, and excellent reproductive capabilities are essential. Body conformation traits are associated with health and fertility, and improvements in these traits can reduce culling rates and facilitate easier calving and insemination, thereby directly impacting subsequent milk production [16]. It has been reported that cows requiring assistance during parturition produce 703 kg less milk, 24 kg less milk fat, and 21 kg less milk protein over a 305-day lactation period compared to cows that calve without assistance [17]. Body conformation traits in dairy cows are economically significant and correlate with calving ease, longevity, locomotion, and milk production traits. The pleiotropic associations between calving performance and rump traits support the known genetic correlation between characteristics of the pelvic area (e.g., rump traits) and calving ease and calf survival. A cow with a wide pin, long sloping rump, and slight slope from the pin bone to thurl is known to have easier calving [18,19]. Additionally, correlations have been observed between body conformation traits such as body condition score (BCS), stature (STA), udder depth (UDE), chest width (CWI), and milk production traits [20].

In this research, we focused on significantly enhancing the accuracy of genetic predictions for key conformation traits in modern Korean Holstein cattle. Our approach involved conducting a comprehensive genome-wide association study (GWAS) on 24 critical traits. Using five SNP panels, our objective was to pinpoint SNP markers, genes, and chromosomal regions linked to these traits across 29 autosomes, thereby enriching our understanding of their genetic underpinnings in Korean Holstein cattle. This study’s findings are crucial in assessing the effectiveness of genomic selection for body conformation traits in Korean Holsteins. Moreover, our results underscore the potential benefits of leveraging larger datasets and more advanced genomic models to markedly improve the precision of breeding strategies, particularly for traits that have significant economic and health relevance.

## 2. Materials and Methods

### 2.1. Phenotype and Genotype Data

We used phenotypic data for the following 24 traits: stature (STA), chest width (CWI), body depth (BDE), angularity (ANG), rump angle (RAN), rump width (RWI), rear leg set (RLS), foot angle (FAN), fore udder attachment (FUA), rear udder height (RUH), rear udder width (RUW), udder support (USU), udder depth (UDE), front teat placement (FTP), front teat length (FTL), overall conformation score (OCS), loin strength (LST), udder texture (UTX), rear teat placement (RTP), heel depth/foot height (HDE), bone quality (BQL), rear leg rear view (RLR), height at the front end (HHE), locomotion (LOC), and body condition score (BCS). Conformation scores were recorded according to the guidelines provided by KAIA. The estimated heritability of the 24 body conformation traits ranged from 0.031 to 0.334 (Table 1). Within the body traits, STA exhibited moderate heritability over 0.30, as did RAN within rump traits and UDE within udder traits.

Genomic data for a total of 11,660 cattle collected from three domestic institutions were compiled using the Illumina and Axiom platforms. This dataset serves as a foundation for analyzing genetic diversity and traits, utilizing the Illumina50Kv2 and Illumina50Kv3 (Illumina, Inc., San Diego, CA, USA), Axiom-BovMDv3, AxiomCustom300K platforms and KHOLs60Kv1 (Affymetrix, Inc., Santa Clara, CA, USA). To ensure the accuracy and reliability of the data, multiple stages of rigorous quality control procedures were applied to SNP markers and animal data.

During the quality control process, unmapped SNPs and SNPs located on sex chromosomes were initially excluded. Subsequently, SNPs with a call rate below 0.95, a minor allele frequency (MAF) below 0.01, and a Hardy–Weinberg equilibrium (HWE) *p*-value below 0.0001 were removed to enhance the quality of the data. Based on these criteria, the number of available SNP markers for each panel (KHOLs60Kv1, Illumina50Kv2 and v3, AxiomBovMDv3, AxiomCustom300K) was determined.

In the next step, genotypic data from various panels were imputed to the AxiomCustom300K level for integration, using the UMD 3.1 mapping information as a reference. This process is essential for maintaining consistency in genotypic data and increasing the accuracy of analyses. The SNP genotyping data for each panel underwent a separate imputation process using FImpute version 2.2 software [21], ensuring that all genetic information was accurately imputed back into their respective panels.

After the quality control and data imputation processes, the final number of animals analyzed per panel and the number of SNPs were determined. These genomic data form a crucial basis for analyzing genetic characteristics and diversity. The insights gained from these processes can be utilized in various research and applications, including genetic improvement, disease resistance, and productivity enhancement in cattle.

### 2.2. Model Definition

#### 2.2.1. Deregressed Estimated Breeding Values (DEBVs) of the Response Variable in Genomic Analysis

Data were obtained from the national genetic evaluation of dairy cattle conducted by the National Institute of Animal Science (NIAS) of the Rural Development Administration (RDA), including variance components and estimated breeding values (EBVs). The variance components considering lactation group and HYS, provided by the National Institute of Animal Science in Korea, were applied to 25 linear-type traits. DEBVs were re-estimated using the estimated individual genetic values, reliabilities, and genetic variance components. DEBVs were re-estimated with the aim of using them as response variables for GS. Because the newly estimated response variables had varying reliability for each individual, a weighting factor was applied to account for this heterogeneous variance using Equation (1) [11]:

(1) $$w_{i}=\frac{\left(1-h^{2}\right)}{[c+(1-r_{i}^{2})/r_{i}^{2}]h^{2}}$$

where $r_{i}^{2}=$ reliability of DEBV response variables including and excluding the parent average (DEBVincPA and DEBVexcPA), $h^{2}=$ heritability of each trait, and c= proportion of genetic variation that cannot be explained by the markers. In this study, c was assumed to be 0.4 [22]. The resulting dataset, consisting of 11,095 reference animals with both genomic and phenotypic (DEBVs) information, was used for a GWAS and a genomic prediction model analysis using Bayesian methods.

#### 2.2.2. Statistical Method for GS Model

In our research, the estimation of SNP marker effects for GWAS and genomic prediction models was conducted using an adapted Bayesian approach [2]. This method involved the application of modified BayesB [2] and BayesC [23] models, which were implemented through the specialized GenSel4R.8R software framework [24]. The approach was distinctive in that it employed a set of varied π values (0.75, 0.90, 0.99, and 0.995), each accompanied by specific weighting factors. Our version of the BayesB model was based on a mixed model concept, hypothesizing that a certain proportion π of SNP markers exhibit null effects, while the remainder display non-zero impacts. This method diverged from standard practices by utilizing a t-distribution as the foundational assumption for SNP marker effects and by factoring in locus-specific variances. The BayesC model is similar to the BayesB model, but it differs in that it assumes all markers contribute to genetic variation, with the effects of all markers sharing a common variance. BayesC additionally models the probability that the variance of marker effects is zero. For each individual trait under examination, we rigorously applied this tailored model to accurately estimate the effects of SNP markers, aligning with our modified equation framework.

(2) $$y_{i}=\mu +\sum_{j=1}^{k}Z_{ij}u_{j}\delta_{j}+e_{i}$$

where $y_{i}=$ response variables (DEBVexcPA and DEBVincPA) for each trait, μ= overall mean, k= number of markers, $Z_{ij}=$ allelic state at the *j*th locus (−10, 0, and 10 in GenSel4R) in the *i*th individual, $u_{j}=$ random substitution effect for the *j*th marker, which follows a mixture distribution for this random substitution effect according to the indicator variable ($\delta_{j}$), a random 0/1 variable indicating the absence or presence of marker j in the model, with $u_{j}$ assumed to be normally distributed $N(0,\sigma_{u}^{2}$) when $\delta_{j}=1$, and otherwise, $u_{j}$ is assumed to be 0; and $e_{i}$ is a random residual effect assumed to be normally distributed $N(0,\sigma_{e}^{2}$).

To ascertain the posterior distributions of these parameters and their effects, we utilized Gibbs sampling in conjunction with a Markov Chain–Monte Carlo (MCMC) process encompassing 110,000 iterations in total. The initial 10,000 iterations were eliminated as part of the burn-in phase to ensure model stabilization. Post this phase, we meticulously gathered 10,000 samples, strategically selecting every tenth iteration to minimize the potential for autocorrelation, a technique referred to as ‘thinning’. These carefully curated samples were pivotal in calculating the posterior means for the effects and variances of the SNP markers.

#### 2.2.3. Genomic Prediction Accuracy under Fivefold Cross-Validation

In this study, we used the K-means clustering method with 5-fold cross-validation to estimate genomic accuracy. K-means clustering divided the reference population into training and validation groups while minimizing the genetic relatedness between these two groups. For the K-means clustering method, we used pedigree data from 54,346 individuals related to the 11,095 individuals used in the genomic analysis to estimate a numerical relationship matrix (NRM). Estimates an NRM and transforms it into a distance matrix to effectively minimize the genetic relationships between groups. The clustering indicated that the statistics a_max_ and a_ij_ within the groups were relatively distant from the statistics a_max_ and a_ij_ between the groups (Table 2). However, notably, K-means clustering did not achieve perfect separation in one clustering group, possibly because of the limited connectivity between domestic cattle and imported individuals in the pedigree analysis.

## 3. Results and Discussion

### 3.1. GWAS of Each Trait

The significance of body conformation traits in dairy cows lies in their association with reproductive and milk production traits. In Chinese Holsteins, STA, BDE, and BQL exhibit strong positive genetic correlations with milk production traits, indicating that cows with greater STA, deeper BDE, and superior BQL tend to produce more milk enriched with higher fat and protein content and possess better somatic cell scores [25]. ANG showed a very high negative genetic correlation with BCS at −0.612, and milk yield and BCS exhibited a correlation of −0.386, suggesting that higher milk-producing cows tend to be leaner [26]. STA demonstrated significant genetic correlations with milk yield (0.34), milk fat percentage (0.30), milk protein percentage (0.32), somatic cell score (0.23), and 305-day milk yield (0.22). RAN was genetically correlated with calving interval at −0.16 [27]. Extensive research on the genetic correlation between body conformation traits and somatic cell scores revealed the strongest associations with FUA, FTP, and UDE [28].

In the GWAS results, we present regions that exhibit a proportion of genetic variance (GV) explained by 1.0 Mb of 1.0% or higher, along with informative SNPs and gene annotation outcomes (Table 3, Figure S1). Assuming an infinitesimal model, the expected proportion of genomic variance explained by each window was 0.04% ([1/2524] × 100). However, for cases with 1.0%, the association is 25 times higher, leading us to set the significance level at GV 1.0%. When a marker accounts for more than 1.0% of the genetic variation, it signals a strong genetic influence on the trait or disease in question. This level of variation is vital for achieving statistically significant results in GWAS, which requires analyzing large datasets. We used the BayesB method with a large value of π with DEBVincPA acting as a response variable for each trait. We opted for the BayesB method due to its superior handling of sparse genetic data and its ability to provide more accurate and flexible analysis of genetic contributions to traits, enhancing our research’s precision and insight. Among the 24 traits examined, 12 exhibited a GV of 1.0% or higher. Therefore, in the main text, we focused solely on traits with a GV exceeding 1.0%, while the remaining traits and research findings are provided in the supplementary materials (Table S1).

For ANG, we identified one informative SNP in a specific region, and gene annotation revealed presence of the *GC* gene. *GC*, responsible for encoding vitamin D-binding protein (DBP), has consistently emerged as the leading candidate gene for clinical mastitis (CM) resistance QTL [29,30]. Furthermore, the application of vitamin D as a therapeutic measure in lactating cows fed CM has been shown to reduce inflammation, implying a potential association between vitamin D and inflammation [31,32,33]. For the BCS trait, we identified eight informative SNPs within a specific region, and annotation revealed the presence of *ANKAR*, *MSTN*, *ASNSD1*, and *OSGEPL1*. Among these, the *MSTN*, *OSGEPL1*, *ANKAR*, and *ASNSD1* genes have been reported to show a strong association with direct calving difficulty in cattle [34] and have been identified as candidate genes for milk yield and composition in dual-purpose Belgian Blue cows [35]. Additionally, the *ANKAR*, *OSGEPL1*, and *ASNSD1* genes are adjacent to markers showing significant effects on the lactose percentage trait [36]. The *MSTN* gene is known to regulate the proliferation and differentiation of skeletal muscle cells, and various gene mutations are associated with skeletal muscle mass in several mammalian species [37,38]. Additionally, muscle weakness has been observed in mice with a knockout of the *ASNSD1* gene [39]. For BQL, we identified seven informative SNPs within three distinct regions. Gene annotation of these regions revealed the presence of *GC*, *ENSBTAG00000046149*, *GDPD5*, *ENSBTAG00000000628*, *MEIS2*, and *TMCO5A*. Notably, the silencing of *GDPD5* has been shown to decrease cell proliferation, migration, and invasion in breast cancer [40]. Additionally, it is considered one of the hub genes influencing milk yield traits in buffaloes [41]. Furthermore, *MEIS2* was associated with carcass traits in Chinese Simmental beef cattle [42]. For CWI, we identified 10 informative SNPs within three distinct regions. Gene annotation of these regions revealed the presence of *DECR1*, *CCT8L2*, *XRCC2*, *KMT2C*, *GALNTL5*, and *CTNNA2*. However, no study has associated these genes with conformation traits. Nevertheless, *CTNNA2* has been reported as a candidate gene for milking speed in North American Holstein cattle [43]. For the FTL trait, we identified one informative SNP within a specific region. Gene annotation of this region revealed the presence of the *FAM49A* gene. The *FAM49A* gene is associated with milk production in German Holstein cattle [44] and also had been reported as one of the candidate genes for milking speed in French Holstein cattle [45]. This gene was additionally associated with rear udder height in Holstein cattle [46]. For FTP, we identified 12 informative SNPs within two distinct regions. Gene annotation of these regions revealed the presence of *SHOX2*, *ENSBTAG00000047546*, and *ZNF608*. The *SHOX2* gene is strongly associated with milk traits and growth and development in dairy goats [47] and is reported to be involved in height and chondrogenesis [48,49]. For HHE, we identified two informative SNPs within a specific region. Gene annotation of this region revealed the presence of *ANKH* and *TRIO*. The *ANKH* gene plays a crucial role in familial and severe forms of sporadic chondrocalcinosis [50]. Furthermore, *ANKH* encodes an inorganic pyrophosphate transport regulator that prevents deposition of calcium and phosphate in bones [51]. For RAN, we identified one informative SNP within one region. The *FCHSD1* gene has been identified in this region; however, no study has associated it with body conformation traits. For RTP, we identified five informative SNPs within two regions. The annotation results for these regions revealed the presence of *DOCK2* and *CA10*. The *DOCK2* gene is associated with immune processes, including lymphocyte trafficking [52]. For RUW, we identified three informative SNPs within one region. The annotation results for this region revealed the presence of the *GC* and *GPC5* genes. Overexpression of *GPC5* is also known to inhibit cell proliferation [53]. For RWI, we identified four informative SNPs within the regions. The annotation results for these regions revealed *IKBKB*, *HELZ*, *CEP112*, and *CACNG5*. The *HELZ* gene is known to promote cell proliferation [54]. For STA, we identified three informative SNPs within a single region. Annotation results for this region revealed that *MACROD2* was located near the markers. The *MACROD2* gene is a candidate gene affecting RUH in Holstein cattle [46]. It is also associated with reduced antral follicle count (AFC) and anti-Müllerian hormone (AMH) in humans [55]. Furthermore, *MACROD2* is associated with survival in dairy cattle [56], resistance to bovine tuberculosis [57], and mediation of cell growth and proliferation in humans [58].

### 3.2. Estimation of Genomic Prediction Accuracy

In this study, we compared genomic accuracy using various π levels (0.75, 0.90, 0.99, 0.995) and two Bayesian methods (BayesB and BayesC). Accuracies were estimated for 12 traits: ANG, BCS, BQL, CWI, FTL, FTP, HHE, RAN, RTP, RUW, RWI, and STA, at π levels of 0.75, 0.90, 0.99, and 0.995 (Table 4 and Table 5). The remaining traits and research findings are provided in the supplementary materials (Tables S2 and S3). We observed a trend where accuracy increased with increasing π levels for certain traits (ANG, BCS, BQL CWI, FTL, FTP, and RTP using DEBVexcPA; RWI using DEBVexcPA) while it decreased for others (HHE and RTP using DEBVincPA;, RUW and RWI using DEBVincPA, STA). The highest accuracy was noted for the RAN trait when DEBVincPA was used as the response variable, ranging between 0.441 and 0.447. Overall, the accuracy improved when using DEBVincPA compared with that using DEBVexcPA for all traits. Similarly, in the BayesC method, accuracy changed with different π levels. We categorized these changes as negligible (ANG, CWI, HHE, and RAN), increasing with increasing π levels (BCS, BQL, FTL, FTP, RTP, and RWI), and decreasing with increasing π levels (RUW and STA). For RAN using DEBVincPA as the response variable, the highest accuracy ranged from 0.445 to 0.451, which was slightly higher than that observed with BayesB.

Although there were observable discrepancies, the distinctions in genomic precision among varied π values did not present significant statistical variance for the traits under study. Notably, it is recognized that BayesC exhibits a heightened sensitivity to the actual distribution of marker effects compared to other Bayesian methodologies [29], suggesting that significant accuracy differences could be expected at various π levels. However, in the present study, we did not find any such differences. The accuracy estimated by BayesB was higher for some traits (BCS, FTL, and HHE using DEBVincPA; RTP using DEBVexcPA, STA), whereas BayesC estimated higher accuracy for others (ANG, BQL, CWI, FTP, and HHE using DEBVexcPA; RAN and RTP using DEBVincPA); in some cases (RUW and RWI), no differences were observed.

BayesB, assuming varied marker variance values, tends to outperform BayesC, which operates under the assumption of uniform marker variance values. Notably, BayesB shows superior effectiveness when an estimated π value is applied. Moreover, a range of factors markedly impacts the precision of genomic predictions [59]. Among these, the choice of response variable is paramount, influenced by the data’s structure and distinct attributes. Earlier research advocated for the use of estimated breeding values (EBV) as a response variable, considering the limited scope of applicable information [14,60]. EBV is often favored over DEBV when its reliability is consistent across all genotyped animals. Omitting PA from EBV is advisable to avoid redundancy and to moderate individual EBV towards the PA [11]. On the other hand, accounting for PA after deregression helps in acknowledging variances in PA among genotyped animals, such as disparities arising from inter-family differences [61].

In our study, we selected DEBV as the response variable and noted enhanced genomic precision when PA was considered. The issue of double counting seemed less critical as our model included both progeny and parental genotypes, reducing genetic linkages among genotyped animals from different family lines. Importantly, we found that body conformation and udder-related traits were associated with milk production, while traits like pelvis width were related to reproductive and calving traits. Therefore, applying a genomic selection model could lead to an increase in genetic improvement. Although heritability may not be high and genomic accuracy might not be as elevated as other milk yield-related economic traits, utilizing genomic information can still boost genetic improvement. This inclusion of PA into DEBV was particularly beneficial for the genomic selection of body conformation traits in Korean Holstein cattle. Our study serves as a pioneering effort in predicting genomic accuracy for these traits, providing a crucial reference for future research. Given the paucity of similar studies, further research involving larger groups of animals is imperative to corroborate these findings in Korean Holstein cattle.

## 4. Conclusions

This study marks a pivotal advancement in genomic prediction accuracy for body conformation traits in Korean Holstein cattle, enriched by key findings from GWAS and an analysis of various π levels (0.75, 0.90, 0.99, and 0.995) along with Bayesian methods (BayesB and BayesC). Our GWAS identified several novel genetic markers that were significantly associated with body conformation traits. These markers are crucial for understanding the genetic underpinnings of these traits and provide a solid foundation for genomic predictions. Notably, specific loci were found to have a profound influence on traits, such as RUW and BCS, offering new insights into cattle genetics. This study also underscores the higher predictive accuracy of DEBVincPA over DEBVexcPA for body conformation traits. This suggests that including PA in DEBV considerably enhances prediction accuracy, which is an important consideration for breeding programs. Furthermore, our investigation revealed that the choice between BayesB and BayesC should be based on the traits and genetic markers identified by the GWAS. This approach helps tailor genomic prediction strategies for specific traits, thereby optimizing the accuracy and effectiveness of selection in breeding programs.

In conclusion, the integration of GWAS findings with genomic prediction methods provides a more nuanced understanding of the genetic factors that influence body conformation traits in Korean Holstein cattle. These insights are not only pivotal for refining cattle breeding strategies but also add valuable knowledge to the field of animal genetics and GS.

## Figures and Tables

**Table 1:** Description, variance components, and genetic parameters of body conformation traits in Korean Holsteins.

Trait	Abbreviation	$\sigma_{a}^{2}$ *	$\sigma_{e}^{2}$ *	$h^{2}$ *
Body Trait				
Angularity	ANG	0.106	0.777	0.120
Body condition score	BCS	0.109	0.474	0.187
Body depth	BDE	0.218	0.605	0.265
Chest width	CWI	0.124	0.671	0.156
Height at front end	HHE	0.027	0.302	0.082
Locomotion	LOC	0.027	0.844	0.031
Overall conformation score	OCS	1.027	5.599	0.155
Stature	STA	0.411	0.873	0.320
Rump Trait				
Loin strength	LST	0.075	0.683	0.099
Rump angle	RAN	0.527	1.190	0.307
Rump width	RWI	0.164	0.806	0.169
Feet and leg traits				
Bone quality	BQL	0.064	0.529	0.108
Foot angle	FAN	0.073	1.017	0.067
Heel depth/foot height	HDE	0.031	0.513	0.057
Rear leg rear view	RLR	0.092	1.073	0.079
Rear leg set	RLS	0.108	0.823	0.116
Udder traits				
Front teat length	FTL	0.328	1.219	0.212
Front teat placement	FTP	0.194	0.934	0.172
Fore udder attachment	FUA	0.178	1.170	0.132
Rear teat placement	RTP	0.077	0.769	0.091
Rear udder height	RUH	0.252	1.239	0.169
Rear udder width	RUW	0.114	0.932	0.109
Udder depth	UDE	0.418	0.833	0.334
Udder support	USU	0.115	0.960	0.107
Udder texture	UTX	0.077	0.769	0.091

*, additive genetic variance, residual variance, heritability.

**Table 2:** Comparison of relationships among animals within and across clusters in a 5-fold cross-validation.

Cluster	No. of Animals	inBreC ^1^	a_max_within_^2^	a_max___between_^3^	a_ij_within_^4^	a_ij_between_^5^
1	1585	0.049	0.504	0.401	0.182	0.095
2	1715	0.018	0.362	0.381	0.045	0.064
3	1840	0.041	0.449	0.456	0.098	0.092
4	2039	0.052	0.516	0.426	0.165	0.100
5	3916	0.049	0.538	0.430	0.159	0.092
Avg.		0.042	0.474	0.419	0.130	0.089

^1^, the average of inbreeding coefficients within cluster; ^2^, the average of a_max_ (the maximum of relationships [a_ij_] for each animal) values within cluster; ^3^, the average of a_max_ values between the clustered (training and validation) groups; ^4^, the average of a_ij_ (relationships) values within cluster; ^5^, the average of a_ij_ values between clustered groups.

**Table 3:** Informative SNPs in the significant 1 Mb windows associated with each trait in Korean Holstein dairy cattle from GWAS using SNP genetic markers from the AxiomCustom300K genotyping platform.

Trait ^1^	BTA _MB ^2^	nSNPs	GV (%) ^3^	Informative SNP	Model _Freq ^4^	Variant	Gene Annotation ^5^
ANG	6_88	18	1.52	AX-106731967	0.026	intergenic	GC (152,138)
BCS	2_6	174	1.39	AX-427902438	0.024	intron	ANKAR
				AX-419793308	0.018	downstream gene	MSTN (4600)
				AX-419783071	0.074	5_prime_UTR	ASNSD1
				AX-372108927	0.021	splice region	OSGEPL1
				AX-320887755	0.055	missense	ASNSD1
				AX-320881517	0.019	downstream gene	MSTN (471)
				AX-310503226	0.050	missense	ASNSD1
				AX-117090049	0.031	intergenic	ASNSD1 (29,106)
BQL	6_88	18	1.72	AX-106731967	0.022	intergenic	GC (152,138)
	15_55	28	1.31	AX-115108455	0.011	intergenic	ENSBTAG00000046149 (6310)
				AX-106751411	0.602	intron	GDPD5
				AX-106734828	0.026	intergenic	ENSBTAG00000000628 (18,676)
	10_33	73	1.16	AX-429460221	0.150	intergenic	MEIS2 (276,619)
				AX-428872678	0.016	intergenic	MEIS2 (236,727)
				AX-428146406	0.039	intergenic	TMCO5A (34,691)
CWI	14_76	53	1.85	AX-169515619	0.015	synonymous	DECR1
				AX-115107389	0.020	missense	DECR1
	4_115	66	1.16	AX-429698651	0.323	intergenic	CCT8L2 (65,651)
				AX-429411702	0.012	intergenic	XRCC2 (35,174)
				AX-429252290	0.024	intron	KMT2C
				AX-429200877	0.397	intron	KMT2C
				AX-428377847	0.021	intron	GALNTL5
	11_52	41	1.00	AX-429187627	0.612	intergenic	CTNNA2 (1,899,780)
				AX-428077681	0.012	intergenic	CTNNA2 (1,912,867)
				AX-181635443	0.036	intergenic	CTNNA2 (2,016,344)
FTL	11_82	44	1.60	AX-429750872	0.890	intergenic	FAM49A (120,784)
FTP	1_110	42	1.43	AX-185114427	0.107	intergenic	SHOX2 (136,580)
				AX-106726281	0.017	intergenic	SHOX2 (13,662)
	7_30	82	1.13	AX-429548887	0.017	intergenic	ENSBTAG00000047546 (303,880)
				AX-428051278	0.272	intergenic	ZNF608 (329,604)
				AX-428025513	0.017	intron	ZNF608
				AX-185121878	0.015	intergenic	ENSBTAG00000047546 (270,407)
				AX-181636085	0.024	intergenic	ENSBTAG00000047546 (194,374)
				AX-124375841	0.168	intergenic	ZNF608 (53,173)
				AX-124374944	0.019	intergenic	ENSBTAG00000047546 (174,952)
				AX-115107130	0.034	intergenic	ZNF608 (85,253)
				AX-106758093	0.020	intergenic	ENSBTAG00000047546 (208,718)
				AX-106731571	0.037	intergenic	ZNF608 (13,676)
HHE	20_58	85	2.25	AX-429574557	0.017	intergenic	ANKH (89,616)
				AX-106750002	0.733	intron	TRIO
RAN	7_54	41	1.04	AX-429464352	0.642	intron	FCHSD1
RTP	6_88	77	1.53	AX-428094398	0.691	intron	DOCK2
	19_1	53	1.42	AX-429832035	0.030	intron	CA10
				AX-428891026	0.018	intron	CA10
				AX-320910317	0.547	intron	CA10
				AX-310532733	0.062	intron	CA10
RUW	6_88	18	2.41	AX-106731967	0.140	intergenic	GC (152,138)
				AX-185111344	0.021	intron	GPC5
				AX-106721808	0.152	intron	GPC5
RWI	27_36	40	1.17	AX-429028592	0.726	intron	IKBKB
	19_63	46	1.12	AX-428805994	0.047	intron	HELZ
				AX-320913575	0.047	intron	CEP112
				AX-115116940	0.068	intergenic	CACNG5 (19,442)
				AX-106744348	0.021	intergenic	HELZ (25,925)
				AX-106720992	0.045	intron	HELZ
STA	13_9	93	1.07	AX-428976408	0.841	Intergenic	MACROD2 (340,366)
				AX-428713125	0.028	Intergenic	MACROD2 (299,032)
				AX-310551137	0.019	intergenic	MACROD2 (546,675)

^1^, angularity (ANG), body condition score (BCS), bone quality (BQL), chest width (CWI), front teat length (FTL), front teat placement (FTP), height at front end (HHE), rump angle (RAN), rear teat placement (RTP), rear udder width (RUW), rump width (RWI), stature (STA); ^2^, *Bos taurus* chromosome; ^3^, percentage of additive genetic variance explained by SNP markers within each 1 Mb window region; ^4^, proportion of Markov Chain–Monte Carlo iterations that included the corresponding SNP marker; ^5^, gene symbols when intronic or gene symbols (distance) adjacent to the marker were intergenic.

**Table 4:** Comparisons of genomic prediction accuracy with standard error among genotyping platforms including AxiomCustom300K according to BayesB method with four different π values in each response variable.

Traits ^1^	resVars ^2^	BayesB (with π)			
		0.75	0.9	0.99	0.995
ANG	DEBVexcPA	0.038 (0.032)	0.040 (0.032)	0.052 (0.031)	0.057 (0.032)
	DEBVincPA	0.126 (0.031)	0.128 (0.031)	0.139 (0.031)	0.144 (0.031)
BCS	DEBVexcPA	0.135 (0.033)	0.136 (0.033)	0.145 (0.033)	0.152 (0.033)
	DEBVincPA	0.218 (0.032)	0.220 (0.032)	0.232 (0.032)	0.237 (0.032)
BQL	DEBVexcPA	0.104 (0.034)	0.105 (0.034)	0.113 (0.034)	0.118 (0.034)
	DEBVincPA	0.177 (0.033)	0.180 (0.033)	0.193 (0.033)	0.195 (0.033)
CWI	DEBVexcPA	0.077 (0.032)	0.077 (0.032)	0.091 (0.032)	0.100 (0.032)
	DEBVincPA	0.156 (0.032)	0.154 (0.032)	0.159 (0.031)	0.159 (0.032)
FTL	DEBVexcPA	0.241 (0.031)	0.245 (0.031)	0.269 (0.030)	0.276 (0.030)
	DEBVincPA	0.328 (0.029)	0.333 (0.029)	0.353 (0.029)	0.356 (0.029)
FTP	DEBVexcPA	0.210 (0.031)	0.214 (0.031)	0.236 (0.031)	0.243 (0.030)
	DEBVincPA	0.293 (0.030)	0.297 (0.029)	0.309 (0.029)	0.309 (0.029)
HHE	DEBVexcPA	0.112 (0.035)	0.113 (0.035)	0.120 (0.035)	0.124 (0.035)
	DEBVincPA	0.186 (0.034)	0.186 (0.034)	0.186 (0.034)	0.184 (0.034)
RAN	DEBVexcPA	0.362 (0.031)	0.366 (0.031)	0.377 (0.030)	0.373 (0.030)
	DEBVincPA	0.445 (0.029)	0.447 (0.029)	0.447 (0.029)	0.441 (0.029)
RTP	DEBVexcPA	0.247 (0.031)	0.248 (0.031)	0.254 (0.031)	0.254 (0.031)
	DEBVincPA	0.281 (0.030)	0.283 (0.030)	0.290 (0.030)	0.288 (0.030)
RUW	DEBVexcPA	0.109 (0.032)	0.108 (0.032)	0.097 (0.032)	0.090 (0.032)
	DEBVincPA	0.182 (0.031)	0.181 (0.031)	0.174 (0.031)	0.170 (0.031)
RWI	DEBVexcPA	0.277 (0.031)	0.278 (0.031)	0.285 (0.030)	0.287 (0.030)
	DEBVincPA	0.339 (0.030)	0.341 (0.030)	0.343 (0.030)	0.340 (0.030)
STA	DEBVexcPA	0.312 (0.036)	0.267 (0.031)	0.260 (0.031)	0.246 (0.031)
	DEBVincPA	0.350 (0.030)	0.350 (0.030)	0.340 (0.030)	0.330 (0.030)

^1^, angularity (ANG), body condition score (BCS), bone quality (BQL), chest width (CWI), front teat length (FTL), front teat placement (FTP), height at front end (HHE), rump angle (RAN), rear teat placement (RTP), rear udder width (RUW), rump width (RWI), stature (STA); ^2^, DEBVexcPA = deregressed-EBV excluding parent average; DEBVincPA = deregressed-EBV including parent average.

**Table 5:** Comparisons of genomic prediction accuracy with standard error among genotyping platforms, including AxiomCustom300K according to the BayesC method, with four different π values in each response variable.

Traits ^1^	resVars ^2^	BayesC (with π)			
		0.75	0.9	0.99	0.995
ANG	DEBVexcPA	0.102 (0.031)	0.101 (0.031)	0.099 (0.031)	0.098 (0.031)
	DEBVincPA	0.158 (0.031)	0.158 (0.031)	0.157 (0.031)	0.158 (0.031)
BCS	DEBVexcPA	0.137 (0.033)	0.138 (0.033)	0.140 (0.033)	0.144 (0.033)
	DEBVincPA	0.218 (0.032)	0.218 (0.032)	0.224 (0.032)	0.230 (0.032)
BQL	DEBVexcPA	0.137 (0.033)	0.137 (0.033)	0.138 (0.033)	0.141 (0.033)
	DEBVincPA	0.176 (0.033)	0.177 (0.033)	0.188 (0.033)	0.196 (0.033)
CWI	DEBVexcPA	0.093 (0.032)	0.093 (0.032)	0.101 (0.032)	0.107 (0.032)
	DEBVincPA	0.170 (0.031)	0.172 (0.031)	0.173 (0.031)	0.171 (0.031)
FTL	DEBVexcPA	0.237 (0.031)	0.239 (0.031)	0.262 (0.030)	0.272 (0.030)
	DEBVincPA	0.325 (0.029)	0.328 (0.029)	0.350 (0.029)	0.356 (0.029)
FTP	DEBVexcPA	0.249 (0.030)	0.249 (0.030)	0.254 (0.030)	0.260 (0.030)
	DEBVincPA	0.301 (0.029)	0.301 (0.029)	0.310 (0.029)	0.313 (0.029)
HHE	DEBVexcPA	0.123 (0.035)	0.123 (0.035)	0.125 (0.035)	0.127 (0.035)
	DEBVincPA	0.170 (0.035)	0.170 (0.035)	0.169 (0.035)	0.170 (0.035)
RAN	DEBVexcPA	0.372 (0.030)	0.372 (0.030)	0.376 (0.030)	0.374 (0.030)
	DEBVincPA	0.448 (0.029)	0.448 (0.029)	0.451 (0.029)	0.445 (0.029)
RTP	DEBVexcPA	0.232 (0.031)	0.232 (0.031)	0.240 (0.031)	0.245 (0.031)
	DEBVincPA	0.280 (0.030)	0.280 (0.030)	0.288 (0.030)	0.290 (0.030)
RUW	DEBVexcPA	0.109 (0.032)	0.108 (0.032)	0.103 (0.032)	0.097 (0.032)
	DEBVincPA	0.182 (0.031)	0.182 (0.031)	0.178 (0.031)	0.176 (0.031)
RWI	DEBVexcPA	0.275 (0.031)	0.276 (0.031)	0.282 (0.030)	0.285 (0.030)
	DEBVincPA	0.338 (0.030)	0.339 (0.030)	0.346 (0.030)	0.346 (0.030)
STA	DEBVexcPA	0.248 (0.031)	0.247 (0.031)	0.241 (0.031)	0.235 (0.031)
	DEBVincPA	0.342 (0.030)	0.342 (0.030)	0.336 (0.030)	0.329 (0.030)

^1^, angularity (ANG), body condition score (BCS), bone quality (BQL), chest width (CWI), front teat length (FTL), front teat placement (FTP), height at front end (HHE), rump angle (RAN), rear teat placement (RTP), rear udder width (RUW), rump width (RWI), stature (STA); ^2^, DEBVexcPA = deregressed-EBV excluding parent average; DEBVincPA = deregressed-EBV including parent average.

## Data Availability

The data presented in this study are available on request from the corresponding author.

## Supplementary Materials

The following supporting information can be downloaded at: https://www.mdpi.com/article/10.3390/ani14071052/s1, Table S1: Informative SNPs in the significant 1 Mb windows associated with body conformation traits in Korean Holstein dairy cattle from GWAS using SNP genetic markers from the AxiomCustom300K genotyping platform; Table S2: Comparisons of the genomic prediction accuracy with standard error among genotyping platforms including AxiomCustom300K according to BayesB method with four different π values in each response variable for traits with GV less than 1.0%; Table S3: Comparisons of the genomic prediction accuracy with standard error among genotyping platforms including AxiomCustom300K according to BayesC method with four different π values in each response variable for traits with GV less than 1.0%; Figure S1: Manhattan plots of genome-wide association analysis (GWAS) based on the Bayesian C (BayesC) method for each trait. Angularity (ANG), body condition score (BCS), bone quality (BQL), chest width (CWI), front teat length (FTL), front teat placement (FTP), fore udder attachment (FUA), height at front end (HHE), rump angle (RAN), rear teat placement (RTP), rear udder width (RUW), rump width (RWI), stature (STA).
