# Supplementary material for: Enhancing Genomic Prediction Accuracy for Body Conformation Traits in Korean Holstein Cattle
Source: Animals (Basel). 2024 Mar 29;14(7):1052. doi: 10.3390/ani14071052 (PMC11011013; doi:10.3390/ani14071052)
Supplement: Supplementary file 1 [file animals-14-01052-s001.zip › Fig S1.pdf]

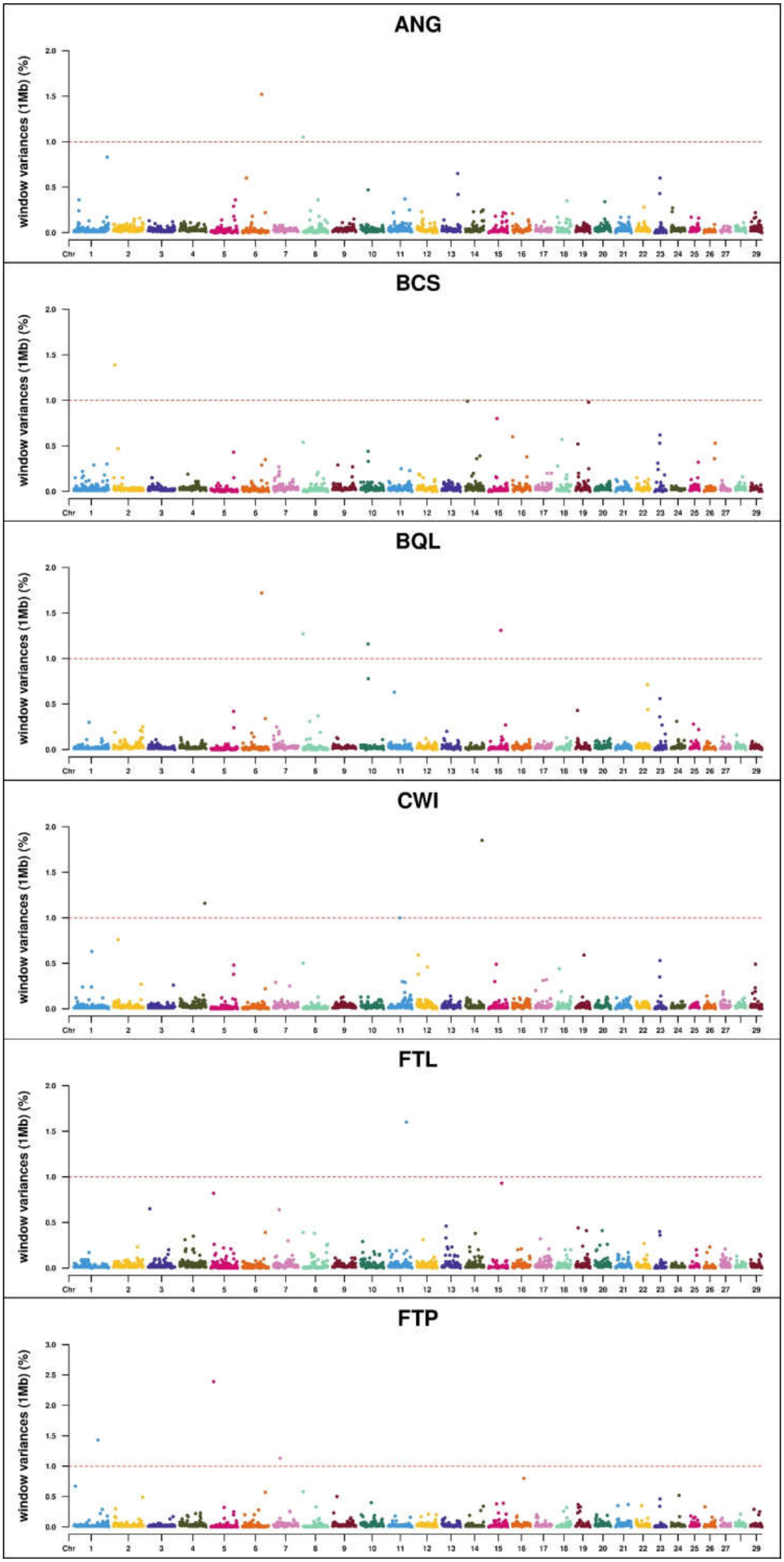

ANG
BCS
BQL
CWI
FTL
FTP
window variances (1Mb) (%)
0.0
0.5
1.0
1.5
2.0
2.5
3.0
Chr
1 2 3 4 5 6 7 8 9 10 11 12 13 14 15 16 17 18 19 20 21 22 23 24 25 26 27 29

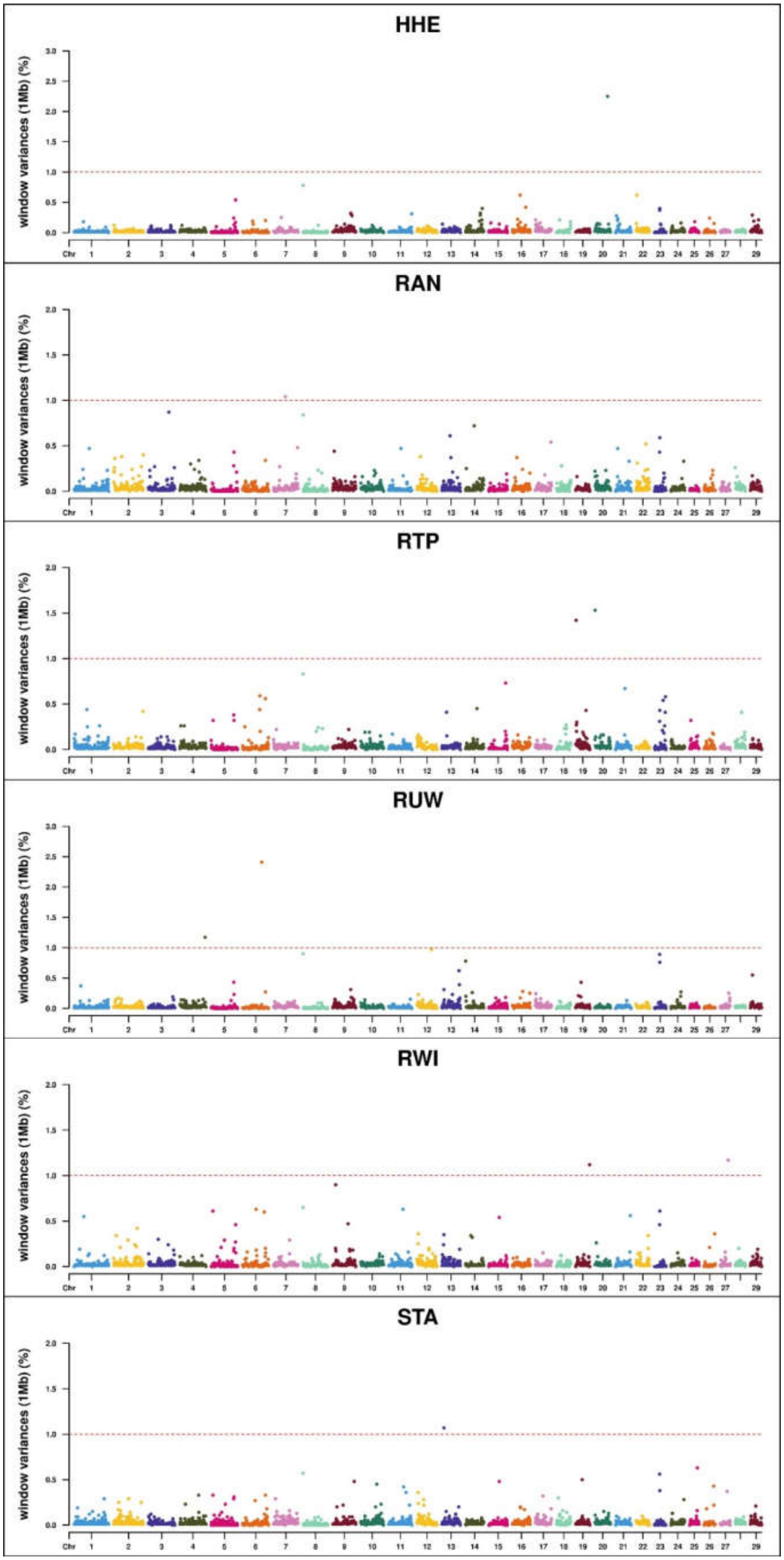


**Figure S1.** Manhattan plots of genome-wide association analysis (GWAS) based on the Bayesian C (BayesC) method for each tratis. angularity (ANG), body condition score (BCS), bone quality (BQL), chest width (CWI), front teat length (FTL), front teat placement (FTP), fore udder attachment (FUA), height at front end (HHE), rump angle (RAN), rear teat placement (RTP), rear udder width (RUW), rump width (RWI), stature (STA)
